# Impact of Asthma Education Meeting on Asthma Control Level Assessed by Asthma Control Test

**DOI:** 10.1097/WOX.0b013e3181c82002

**Published:** 2010-01-15

**Authors:** Ayşe Baççıoğlu Kavut, A Füsun Kalpaklıoğlu

**Affiliations:** 1 The Kirikkale University Faculty of Medicine, Department of Allergic Diseases, Kirikkale, Turkey

**Keywords:** asthma, asthma control level, asthma control test, education, quality of life, SF-36

## Abstract

**Background:**

The asthma control test (ACT) is a reliable tool to measure the level of asthma control. In our research, we aimed to investigate the effect of participation in an asthma awareness session on the patient's perception of current asthma control as evaluated by ACT in relation to quality of life (QoL) in an adult population.

**Methods:**

An observational study in subjects who were diagnosed as suffering with persistent asthma was performed. All asthmatic patients who were followed up in healthcare centers around the city were invited to the study. Patients who consented were informed about the study and then skin prick tests, pulmonary function tests, and blood analyses were performed. In addition, a self-administered generic QoL questionnaire (SF-36) was completed. Finally, the patients were invited to attend the asthma awareness session, and pre-post educational ACT assessments were evaluated.

**Results:**

Overall asthma control was less than optimal in almost half of the study group. ACT level changed in 70.5% of the patients. The change in asthma control by using ACT was prominent in the ACT-deteriorated group than the ACT-improved group (-3.8 ± 2.7 and 2.1 ± 1.3, respectively; *P *= 0.001). Regarding comorbidities, the ACT-deteriorated group had the highest prevalence of rhinitis (*P *= 0.04). The impairment in QoL was similar between the groups and the physical domains of SF-36 were correlated with the ACT scores. The correlation between education level and asthma control was found to be significant after the training session (r = 0.353, *P *= 0.04).

**Conclusion:**

Education in asthma is an essential strategy not only to achieve awareness of asthma control level as assessed by ACT, but also for the reliability of QoL measurement.

## 

Asthma is a serious health problem throughout the world and the goal of asthma therapy is to gain control and improve quality of life (QoL) according to the updated GINA guideline.[1] In 2004, the asthma control test (ACT) was developed to measure asthma control and for self-assessment as part of a written personal asthma action plan.[2] Recently, ACT has been shown to be directly correlated with QoL, as it includes questions about symptoms and daily activities [3].

The majority of patients may not gain an optimal asthma control even with the best medical therapy.[4] Some of the reasons for treatment failure were identified as a lack of awareness, which resulted in an increase in the hospitalization rates, unscheduled visits to the emergency room for reasons of asthma, days missed at work, and nocturnal awakenings.[5] The ideal of informing every patient about the chronic nature of asthma in each visit may not fit with real life in a busy outpatients clinic with limited time and resources. Furthermore, some patients may have difficulties complying with advice that may be helped through peer support group training in addition to education provided by the healthcare provider [6].

Although ACT was not developed as a measure of asthma knowledge, education might be important in the degree of patient understanding. The present trial was set up to test the hypothesis that an asthma education session focused on suitable information about asthma would result in an improved awareness of asthma control in a short time using ACT.

## Materials and methods

Asthma patients were recruited to participate in this observational study in Kirikkale University (tertiary) from randomly selected health centers located in diverse areas of Kirikkale City. Health centers were selected randomly using a computer-based random number generator from a number labeled list of centers. Eligibility criteria were female/male subjects between ages of 18 -60 years with a prescription and doctor diagnose of persistent asthma and with no other respiratory, infectious, or chronic systemic diseases.

In the first visit, patients were asked about sociodemo-graphic characteristics, asthma history, and comorbidities. Education level was classified as low (< 5 years), moderate (6-11 years) and high (> 12 years) according to Turkey standards. Skin prick tests (SPTs) with a battery of common inhalant allergens (ALK, Madrid-Spain), pulmonary function tests (PFTs) (Sensor Medics-2130 Corp.), and blood analysis for total immunoglobulin E (IgE) (ECLIA, Roche) were performed. Subjects were instructed to self-administer a generic QoL questionnaire in the same visit, as well. The after week, patients were participated to the asthma awareness session.

Atopy was accepted if positive SPTs results were clinically relevant. Diagnosis of asthma was confirmed by an allergy specialist, and severity/control level of asthma was assessed as described in GINA [1].

The asthma awareness session given by the allergy specialist consisted of verbal and visual fragments including information about asthma disease, management, avoidance of triggers, and asthma control tools including ACT, interactively. The session that lasted ~2 hours was supplemented by educational slide material provided from Turkish Thoracic Society (TTS) Website http://www.toraks.org.tr/ and ACT forms were also obtained from TTS. Patients fulfilled ACT forms before and 2 hours after the session. The level of asthma control was assessed uncontrolled if ACT score was below 19 or less as mentioned in the original ACT survey study.[2] Medical outcomes study 36-item short form (SF-36) was used to evaluate QoL that had been validated into Turkish language.[7,8] SF-36 assesses 8-health concepts including physical and mental components, and higher scores correspond to a better health status.

The study was approved by the local ethics committee and written informed consents were obtained from the participants. Statistical analysis was performed using SPSS statistical software (Chicago, IL) with a statistically significant *P *value less than 0.05. ACT results, before and after the session were compared by using Wilcoxon Test. Patients were grouped as ACT_-unchanged _(ACT_-before _= ACT_-after_), ACT_-improved _(ACT_-before _< ACT_-after_), and ACT_-deteriorated _(ACT_-before _> ACT_-after_). Group comparisons were done by using Kruskal-Wallis Test for quantitative variables, and by using *χ*^2^/Fisher's exact test for categorical variables. When a cell count was less than 5 in tables with categorical variables, the groups were united. Correlation analyses were performed with Pearson Correlation. Cohen's Kappa statistic was used to calculate agreement between the level of asthma assessed by physician and ACT scores, and kappa values greater than 0.41 are considered to represent moderate agreement [9].

## Results

A total of 44 subjects with a mean age of 41.2 ± 13.8 years (75% of whom were female) were evaluated. Patients were similar in means of age, sex distribution, education level, smoking status, and asthma characteristics (Table 1). After the asthma awareness session, ACT scores changed in 70.4% of patients; 38.6% of them had improved (ACT_-improved_: n = 17), 31.8% had deteriorated (ACT_-deteriorated_: n = 14) and 29.6% had ACT scores unchanged (ACT_-unchanged_: n = 13) (*P *= 0.70). Although almost half of the patients had uncontrolled asthma level before the session, 78.6% of the ACT_-deteriorated _group was found to be uncontrolled after the session (Table 2). Mean change in ACT scores were 2.1 ± 1.3 in the ACT_-improved _group and -3.8 ± 2.7 in the ACT_-deteriorated _group (*P *= 0.001). After the session, mean scores of the ACT-deteriorated group decreased significantly than the other groups. ACT scores were found to be significantly correlated with education level only after the session (r = 0.353, *P *= 0.04). The frequency of severity, exacerbation and hospitalization rates of asthma, and comorbidities including total IgE levels were similar between the groups, except rhinitis with a higher prevalence in the ACT_-deteriorated _group (*P *= 0.04) and longest duration of asthma symptoms in the ACT_-unchanged _group (*P *> 0.05). Neither asthma medications nor predicted values of FEV1 were significantly different between the groups.

**Table 1:** Demographic and Clinical Data of the Patients

N (%)	All Patients (n:44)
Age*	41.2 ± 13.8
Female/male	33 (75)/11 (25)
Education level; (year)*	7 ± 3.5
Low educated	25 (56.8)
Moderate-high educated	19 (43.2)
Current smokers	7 (15.9)
Duration of asthma (year)*	8.9 ± 3.5
Severity of asthma	
Mild persistent	19 (43.2)
Moderate-severe persistent	25 (56.8)
Asthma exacerbation frequency (/yr)*	2.1 ± 1.9
Hospitalisation frequency (/yr)*	0.6 ± 1.0
Asthma comorbidity	
Obesity	23 (52.2)
Drug allergy	5 (11.4)
Gastro esophageal reflux	5 (11.4)
Sinusitis	26 (59.1)
Rhinitis	29 (54.5)
Atopy	14 (31.8)
FEV_1_%*	84 ± 14.5
Asthma management	
LTRA	23 (52.2)
Inhaled steroid	40 (91)
Nasal steroid	18 (40.9)
Long-short acting β_2 _agonist	35 (79.5)
Specific immunotherapy	3 (6.8)
Total IgE (kU/l)*	350 ± 317

*Mean ± SD.

ACT, Asthma control test; FEV_1_, Forced expiratory volume at 1st second; IgE, Immunoglobulin LTRA; Leukotriene receptor antagonist.

**Table 2:** The Effect of the Education on ACT Scores and How They Changed, as Well as the Quality of Life Assed by SF-36 (Uncontrolled ACT is < 20)

N (%)	All (n:44)	ACT_improved _(n:17)	ACT_unchanged _(n:13)	ACT_deteriorated _(n:14)	*P *Value
ACT_before_*	18.1 ± 4.2	17.6 ± 3.8	18.6 ± 4.1	18.1 ± 5.1	0.80
Uncontrolled ACT_before _	24 (54.5)	10 (58.8)	6 (46.2)	8 (57.1)	0.76
Controlled ACT_before _	20 (45.5)	7 (41.2)	7 (53.8)	6 (42.9)	0.76
ACT_after_*	17.6 ± 4.9	19.7 ± 3.6	18.6 ± 4.1	14.2 ± 5.6	0.02
Uncontrolled ACT_after _	25 (56.8)	8 (47.1)	6 (46.2)	11 (78.6)	0.13
Controlled ACT_after _	19 (43.2)	9 (52.9)	7 (53.8)	3 (21.4)	0.13
Mean changes in scores*	0.5 ± 0.7	2.1 ± 1.3	--	-3.8 ± 2.7	0.001
SF-36 scores*					
Physical CS	59.7 ± 23.2	52.5 ± 27.3	62.1 ± 25.1	65.9 ± 16.1	0.51
Mental CS	68.4 ± 51.1	47.9 ± 28.5	68.8 ± 22.2	80.8 ± 76.1	0.14

*Mean ± SD.

ACT, Asthma Control Test; CS, Component summary; SF-36, Short Form Health Survey with 36-item.

The impairment in QoL using SF-36 were found to be similar in all groups when compared with Turkish national norms (*P *> 0.05).[10] However, physical components of SF-36 were correlated with both ACT_-before _and ACT_-after _scores (r = 0.49, *P *= 0.01 and r = 0.50, *P *= 0.01).

The association of ACT and doctor's assessment of the disease control increased from 77.2 to 84.1% after the asthma education meeting, accompanied by a moderate degree with a kappa value (before: 0.549 and after: 0.686).

## Discussion

The present study showed that asthma control was less than optimal in almost half of the asthmatic patients whom were middle aged, low educated with a female dominance. Asthma awareness session resulted to a change in 70.4% of ACT results that might be explained by each person's ability to perception that might vary because of their willingness, mind skills, and training level.

Among the factors that could be related to the patients' understanding of ACT, sociodemographic properties, exacerbation-hospitalization rates, medication, comorbidities were comparable between the groups. ACT_-deteriorated _group had the highest prevalence of concomitant rhinitis and also had the highest uncontrolled asthma ratio that was concordant with previous reports that rhinitis was associated with worse asthma control.[11,12] Misinterpretation of ACT might be related with comorbid diseases in asthmatics as a difficulty to distinguish asthma symptoms from the other system symptoms.

Although, effect of education on QoL was pointed out using various questionnaires,[13-16] this was the first study that evaluated the relation between SF-36 and ACT. The impairment in QoL was shown to be similar among the groups, and ACT was related with almost all physical domains of SF-36. Our previous findings showed that all asthmatic subjects had significantly greater impairment in physical measures.[13] Likewise; Ehrs et al reported a correlation between physical domains of SF-36 with asthma control questionnaire.[14] In this study, SF-36 wasn't used to measure changes after asthma meeting that it was not a measure of acute changes but rather an overall measure of disease state and functionality over time.

The association of ACT and doctor's assessment of the disease control increased after the asthma education meeting. Group session has advantages of both visual and verbal education, and gives an opportunity for questions and learning tips from the answers. Furthermore, the correlation between education level and asthma control may indicate that the awareness session can improve ACT scores, in consistent with the literature [6,17,18].

This study not only showed the importance of education session to recognize the awareness of asthma control, but also that ACT was a reliable tool to measure the changes in asthma control over the time which was also related with physical domains of QoL. Therefore, improvement of awareness in asthma had an effect on the perception of the different domains of ACT and it is important as it reflects the real state of the patient's asthma control. Finally, to achieve the awareness of asthma control level assessed by ACT and to improve QoL, education including both personalized approach and group intervention should be tailored to every asthma patients' follow-up irrespective of asthma control and education level as well.
